# ViralPrimer: a web server to monitor viral nucleic acid amplification tests’ primer efficiency during pandemics, with emphasis on SARS-CoV-2 and Mpox

**DOI:** 10.1093/bioinformatics/btae657

**Published:** 2024-11-05

**Authors:** Norbert Deutsch, Zsuzsanna Dosztányi, István Csabai, Anna Medgyes-Horváth, Orsolya Anna Pipek, József Stéger, Krisztián Papp, Dávid Visontai, Gábor Erdős, Anikó Mentes

**Affiliations:** Department of Biochemistry, Institute of Biology, ELTE Eötvös Loránd University, Budapest H-1117, Hungary; Department of Biochemistry, Institute of Biology, ELTE Eötvös Loránd University, Budapest H-1117, Hungary; Department of Physics of Complex Systems, Institute of Physics and Astronomy, ELTE Eötvös Loránd University, Budapest H-1117, Hungary; Department of Physics of Complex Systems, Institute of Physics and Astronomy, ELTE Eötvös Loránd University, Budapest H-1117, Hungary; Department of Physics of Complex Systems, Institute of Physics and Astronomy, ELTE Eötvös Loránd University, Budapest H-1117, Hungary; Department of Physics of Complex Systems, Institute of Physics and Astronomy, ELTE Eötvös Loránd University, Budapest H-1117, Hungary; Department of Physics of Complex Systems, Institute of Physics and Astronomy, ELTE Eötvös Loránd University, Budapest H-1117, Hungary; Department of Physics of Complex Systems, Institute of Physics and Astronomy, ELTE Eötvös Loránd University, Budapest H-1117, Hungary; Department of Biochemistry, Institute of Biology, ELTE Eötvös Loránd University, Budapest H-1117, Hungary; Department of Physics of Complex Systems, Institute of Physics and Astronomy, ELTE Eötvös Loránd University, Budapest H-1117, Hungary

## Abstract

**Summary:**

Accurate pathogen identification is crucial during outbreaks, especially with the emergence of new variants requiring frequent primer updates. However, resources for maintaining up-to-date verification of primer sequences are often limited, which poses challenges for reliable diagnosis and hinders potential monitoring efforts based on genome sequencing. To address this, we introduce ViralPrimer, a web server facilitating primer design, SARS-CoV-2 and Mpox variant monitoring, and adaptation to future threats. ViralPrimer aims to enhance diagnostic accuracy with its comprehensive primer database, mutation analysis assistance, and user primer upload feature. Its adaptable design allows monitoring of other rapidly mutating pathogens, contributing to broader public health protection efforts.

**Availability and implementation:**

ViralPrimer is freely accessible and open to all users with no login requirement at https://viralprimer.elte.hu/. The application is hosted on a DJANGO v3.2.13 web server, with a PostgreSQL database, and the frontend was implemented using jQuery v3.6.0, vanilla JavaScript vES6, and Bootstrap v5.1.

## 1 Introduction

The importance of accurate diagnostics in comprehending and managing rapidly mutating viral pathogens has become increasingly evident, particularly in the context of the SARS-CoV-2 pandemic, which presents a challenge to the effectiveness of diagnostic techniques (Fernandes *et al.* 2022). Although reverse transcription-polymerase chain reaction (RT-PCR) is currently considered the gold standard for diagnosing SARS-CoV-2, other nucleic acid amplification tests (NAATs) such as loop-mediated isothermal amplification (LAMP) offer rapid, robust, and cost-effective alternatives (Kashir and Yaqinuddin 2020). Additionally, next-generation sequencing (NGS) can be used not only for detecting viral presence (Bloom *et al.* 2021) but also for tracking the evolution of SARS-CoV-2 to identify emerging variants (Rosenthal *et al.* 2022). Consequently, these diagnostic methods as well as sequencing technologies require continuous evaluation, and the tracking of mutations in the target regions of the primers is critical for the reliable and accurate detection and sequencing of the viral genome.

It was shown that mutations in primer target regions can impact binding efficiency, potentially leading to false negative diagnostic results (Ai *et al.* 2020, Chen *et al.* 2020, Li *et al.* 2020a,b, Mentes *et al.* 2022). A recent study estimates that up to 29% of tests could result in misdiagnosis, particularly in highly affected populations (disease prevalence up to 50%), highlighting the importance of careful primer design (Pecoraro *et al.* 2022). While diagnostic primers are crucial for avoiding misdiagnosis, sequencing primers play a vital role in obtaining high-quality genome assemblies for tracking viral evolution. However, the potential impact of primer failure differs between these methods: diagnostic errors have more immediate epidemiological consequences, while errors in sequencing may result in “dropout” or the loss of an amplicon, leading to incomplete genome sequences and lower-quality genomic data (Sanderson and Barrett 2021, Bei *et al.* 2022). The latter is exemplified by the revisions of the ARTIC network and Midnight sequencing primers in response to the emergence of new variants such as Delta or Omicron (Bei *et al.* 2022, Lambisia *et al.* 2022, Ulhuq *et al.* 2023).

Given the importance of accurate primer design to accommodate viral evolution, the lack of readily available and current resources poses a significant challenge. To address this, we propose ViralPrimer, a website to help with primer design, monitor SARS-CoV-2 variants, and adapt to future viral threats. The web server provides an extensive database of diagnostic, sequencing, and LAMP primers and facilitates analysis through visualizations of genomic regions. The variant data is obtained from GISAID (https://gisaid.org/) (Khare *et al.* 2021) and the COVID-19 Data Portal (http://www.covid19dataportal.org) (Cantelli *et al.* 2021), which can be filtered temporally, across various countries of sample origin and by mutational profiles based on Variants of Concern (VOC) lineages. Users can also upload their own primer sequences. The potential of this tool lies in the identification of mutations that may affect primer binding efficiency. Mutations in the primer target regions are classified as high-risk mutations if they occur at the 3ʹ end of the primer or is an indel, and as moderate-risk if they have a minor effect on binding, however, the presence of three or more moderate-risk mutations in a primer's target region can also disrupt binding, although careful detection thresholds and multiple primer systems can mitigate this issue.

ViralPrimer not only enhances the ability to respond effectively to the evolving SARS-CoV-2 landscape but also prepares for future viral threats. Its adaptable design allows monitoring of other rapidly mutating viruses, as demonstrated by the integration of Mpox data.

## 2 Server description

ViralPrimer utilizes DJANGO's third major release (version 3.2.13) for backend functionality. Information is stored in a consistent relational database under the PostgreSQL framework. Data is either fetched directly from the database or computed in real-time from available information, eliminating the need for external APIs. The frontend combines DJANGO template language, jQuery (version 3.6.0), vanilla JavaScript (ES6), and Bootstrap (version 5.1) for its design. The visual elements are predominantly created using jQuery and vanilla JavaScript, with Plotly.js (version 2.11) utilized for chart rendering. Despite its reliance on advanced web technologies, ViralPrimer ensures compatibility across all browsers that support HTML5 and WebP.

## 3 Web interface

ViralPrimer is accessible online at https://viralprimer.elte.hu/, offering an array of user-friendly options for data retrieval. The homepage presents a broad overview of the server's capabilities, including direct links to introductory guides and example use cases. Navigation is facilitated through eight primary tabs: *Home*, *Search*, *Browse*, *Primer mapping*, *Mutation Analysis*, *Help/About*, *Statistics*, and *Tutorials*. The *Search* tab enables users to investigate mutations in a selected reference genome region with advanced filtering options, including collection date (the date the sample was collected), variant (the viral variant present in the sample), country (the geographical location where the sample was collected), and methods (whether the primer is used for sequencing or diagnostic purposes). The *Browse* page provides direct links to related entries, along with their labels and unique identifiers and also presents tables grouping data based on specific attributes. The *Primer Mapping* tab enables users to upload their designed PCR primer sequences to check for mutations in a specific genomic region. The *Mutation Analysis* tab allows users to input multiple positions or mutations, either at the amino acid or nucleic acid level, and assess their potential impact on selected primers for a given pathogen. For more refined search, the advanced filtering offers similar options to those in the Primer Mapping tab. The *Help/About*, *Statistics* and *Tutorials* pages are designed to help users navigate the server by providing examples, data-related information and statistics.

We also present a visual analytics framework within the ViralPrimer that can help users to explore the relationship between primer design and genomic mutations. This framework comprises five key components: *Header*, *Simple Genome Viewer*, *Detailed Region Viewer*, *Mutations in Primer Target Regions*, and *Summary Plots*. The *Header* offers essential contextual information and interactive features for navigating genomic regions. The *Simple Genome Viewer* provides an overview with zoom and navigation functions, delivering detailed primer and gene information. The *Detailed Region Viewer* offers a magnified view of genomic regions, outlining genes, proteins, primers, and mutations. The *Mutations in Primer Target Regions* section identifies and classifies mutations within primer target regions, aiding primer design. The *Summary Plots* segment offers graphical summaries of mutation distributions, allowing temporal and geographic analyses of mutation trends of the selected genomic region.

## 4 Data source

SARS-CoV-2 mutation data was obtained from GISAID (https://gisaid.org/) (Khare *et al.* 2021) and the COVID-19 Data Portal (http://www.covid19dataportal.org) (Cantelli *et al.* 2021, Harrison *et al.* 2021, Rahman *et al.* 2024), using high-quality samples with confirmed mutations [ENA project: PRJEB43947], a process extensively detailed in our previous article (Mentes *et al.* 2022). The Kooplex collaboration platform (https://k8plex-veo.vo.elte.hu/hub/) (Visontai *et al.* 2019) was used to investigate variants and samples from systematically analysed raw reads. Briefly, to ensure high quality, human samples with an estimated N-content of no >10% were selected. Genomic positions with a sequencing depth below 100 and an alternate allele frequency below 0.5 were excluded from analysis. Mpox data was processed following a similar procedure, undergoing systematic analysis of raw read data accessible through the COVID-19 Data Portal to produce a standardized set of variant calls (in VCF format) and consensus sequences [ENA project: PRJEB55823].

The reference sequences and feature data for the virus were obtained from the GenBank file, using the NC_045512.2 reference (Wu *et al.* 2020) [identical to MN996528.1 used by GISAID as the reference (https://gisaid.org/wiv04/) (Zhou *et al.* 2020)] of SARS-CoV-2 and the NC_063383.1 reference (Mauldin *et al.* 2022) of the Mpox genomes. References and features data are available for download under the *Genes* tab of the *Browse* section on ViralPrimer.

In our previous investigation (Mentes *et al.* 2022), we collected numerous primers from reputable scientific literature, which were designed for both traditional and RT-qPCR methods to detect SARS-CoV-2. We have since expanded the repertoire of primers within the ViralPrimer web server by incorporating additional primer systems, including sequencing and LAMP techniques. Additionally, the server hosts diagnostic and sequencing primers designed for the detection of Mpox. Primer data are available for download at the *Primers* tab of the *Browse* section of ViralPrimer. Mutation data can be downloaded from the *Download* section on the *Help/About* page.

## 5 Result and discussion

**Figure 1.**
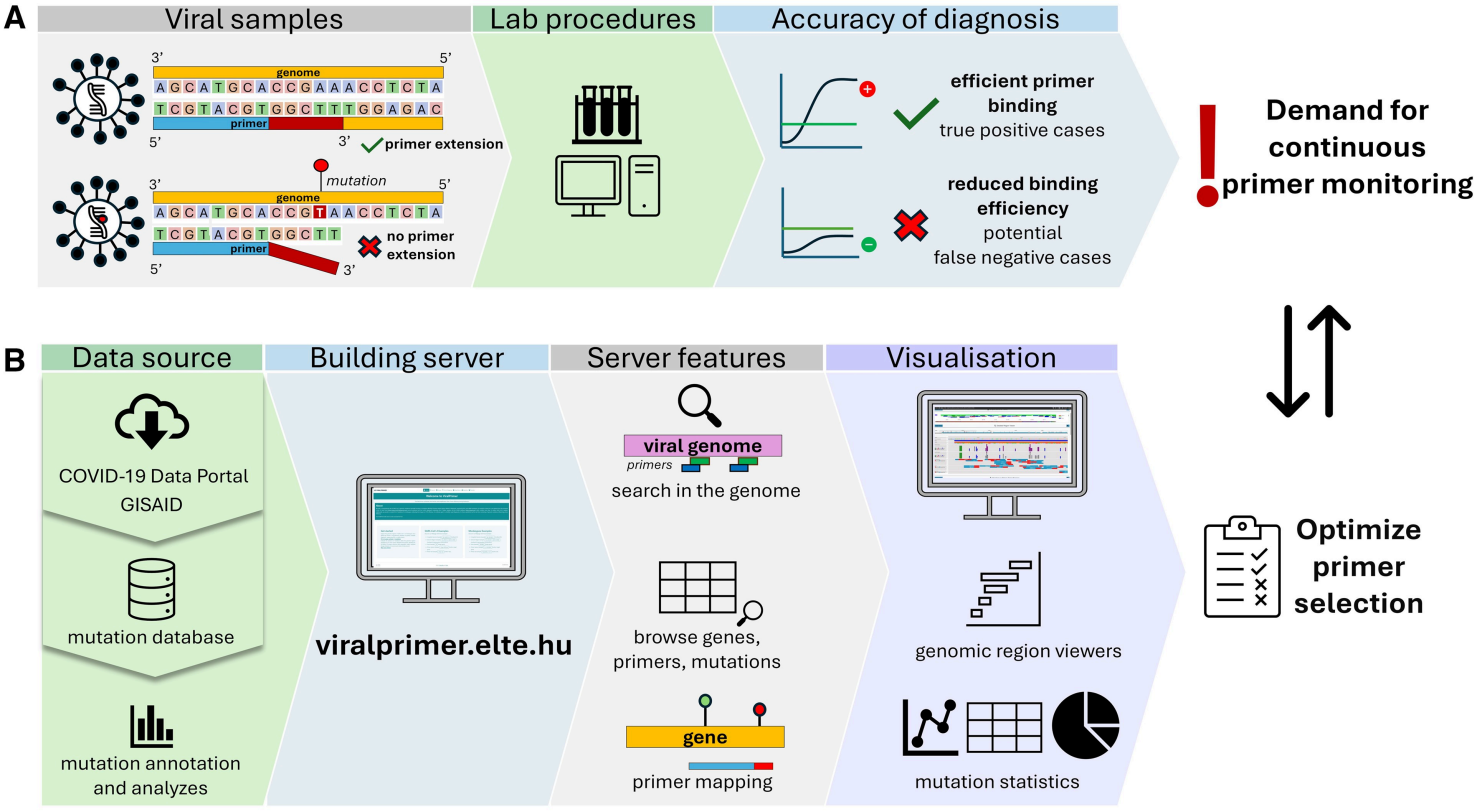
Overview of the ViralPrimer project. (A) The efficiency of PCR tests is affected by the variability in viral genome. Mutations near the 3ʹ end of the primer target region (designated as high-risk) can lead to false-negative results, highlighting the significance of continuous primer assessment. (B) Our project uses data from a mutation database incorporating the analysis results of various public sample sets (GISAID, COVID-19 Data Portal), enabling the assessment of mutations and samples concerning primer binding sites. Classification relies on the genomic location, length, and co-occurrence of mutations in each primer target region. The main features of the server are the options of searching in the genome, browsing primers, and primer mapping. The ViralPrimer webserver offers visualization tools, such as region viewers and mutation statistics, to aid in obtaining results and selecting optimal primers for specific regions. This tool further addresses the ongoing need for rapid primer testing and ensures the accuracy of diagnostic results.

The primary goal of our web server is to integrate genomic mutation data of prevalent global pandemic pathogens with up-to-date information, including VOC variants, geographic locations and date of collection, and sequence information of publicly available PCR, LAMP and sequencing primers (Fig. 1). Previously, various websites have been created to track SARS-CoV-2 variants and monitor mutations in primer binding regions (Primer-monitor, Campbell *et al.* 2021, CoV2ID, Carneiro *et al.* 2020, CoVrimer, Vural-Ozdeniz *et al.* 2021, AssayM, Naeem and Pain 2020). However, some of these platforms are no longer available a few years after the start of the COVID-19 pandemic, host outdated data or require a registration (such as PrimerChecker at GISAID.org). Therefore, a comprehensive, sustainable, and responsive platform is needed to address these challenges. This is crucial not only for efficiently tracking the evolution of pathogens like SARS-CoV-2 but also for swift adaptability to emerging pandemic pathogens to effectively monitor future viral threats in diagnostics.

The ViralPrimer web server has a comprehensive collection of NAATs primers, along with their associated mutations. We collected a total of 1372 primer oligos from 50 primer sets (10 LAMP sets, 4 sequencing sets, 35 diagnostic PCR sets). For SARS-CoV-2, a total of 19 007 636 samples were processed from two databases (GISAID—16 160 967 and COVID-19 Data Portal—2 846 669), while for Mpox, 1574 samples were included. The features allow for quick verification of mutations that affect both public and custom primers. Case studies demonstrating the use of the ViralPrimer website are available on the *Tutorials* page: one investigates the impact of Omicron XBB.1.5 variant mutations (*Use Case 1*), another focuses on advances in SARS-CoV-2 whole genome sequencing and evaluates the effectiveness of long-range PCR primers (Kandel *et al.* 2024) (*Use Case 2*), and a third provides a guide to using the Mutation Analysis page with Mpox amino acid mutations as an example (Wang *et al.* 2023) (*Use Case 3*). The data in the web server is regularly updated to ensure that it contains the most current information. In the future, we plan to expand the ViralPrimer to include viruses that are of global concern.

The ViralPrimer could fill a critical gap in the ability to respond rapidly to emerging viral threats by providing up-to-date and user-friendly resources for primer design and tracking viral evolution.

## Data Availability

The data underlying this article are freely available on the ViralPrimer website at https://viralprimer.elte.hu/. Mutation data used on the website can be downloaded via https://viralprimer.elte.hu/help#download. Detailed NAATs' primer information is accessible at https://viralprimer.elte.hu/browse/primers. Primary data are sourced from the GISAID repository (https://gisaid.org/, with sample authors acknowledged) and the COVID-19 Data Portal (http://www.covid19dataportal.org, ENA projects: PRJEB43947, PRJEB55823). Reference sequences and feature data for SARS-CoV-2 and Mpox were obtained from GenBank (NC_045512.2 and NC_063383.1).
